# Bright’s Disease

**Published:** 1892-01

**Authors:** 


### "BRIGHT'S DISEASE."

In the *American Journal of the Medical Sciences* for October last an exceedingly valuable contribution is made by Dr. Francis Delafield, of New York, to the classification of the Diseases of the Kidney. Dr. Delafield has devoted years of patient study to these diseases, and his classification, the outcome of his labor, is both rational and convenient, and well worthy of adoption.

He proposes to discard the term "Bright's Disease," as misleading, and to classify renal diseases according to the nature of the morbid processes. There are three morbid conditions to which the kidney is subject—congestion, degeneration and inflammation.

Congestion of the kidney is an accumulation of blood in the veins and capillaries of the organ, and depends upon causes which affect the circulation.

Degeneration of the kidney is a secondary process, produced by inorganic poisons, as arsenic, or the poisons of infectious diseases, etc. The only change is in the renal epithelium, which becomes swollen and granular, perhaps disintegrated.

Inflammation of the kidney is attended with three essential features: an escape of the elements of the blood from the vessels, a formation of new tissue, and a death of tissue. The inflammations of the kidney are subdivided into: 1, acute exudative nephritis; 2, acute productive or diffuse nephritis. 3, chronic productive or diffuse nephritis, with exudation; 4, chronic productive or diffuse nephritis, without exudation. There are also suppurative and tubercular nephrites. Exudative inflammation is of short duration, leaving behind no permanent changes in the parts affected, and can be favorably affected by treatment. Productive inflammation runs an acute, sub-acute or chronic course, and effects permanent changes in the inflamed parts.

Such a classification as the above brings with it a rational system of therapeutics. Acute congestion of the kidneys can be relieved by the application of heat to the surface of the body. Acute and chronic degeneration are conditions which we are unable to treat. In acute exudative and acute diffuse nephritis the indication is to diminish the severity of the nephritis and to regulate the circulation. For the former, cups or heat are employed over the lumbar region or the entire body, and calomel, sulphate of magnesia, opium, aconite or digitalis are used internally. The disturbances of the circulation are the causes of the dropsy and cerebral symptoms. With a laboring heart and contracted arteries drugs should be used which dilate the arteries—chloral hydrate, nitrite of amyl, nitroglycerine—or the quantity of blood may be diminished by venesection, sweating or purging. With a feeble heat and relaxed arteries cardiac stimulants are indicated.

In chronic nephritis, climate and mode of life constitute the important parts of the treatment; it is doubtful if drugs exert any effect in the nephritis. A warm, dry climate and an outdoor life are of the greatest importance. Medical treatment may be employed with advantage for the relief of the anæmia, the dropsy and the disturbances of the circulation.

---

The Second Cuban Medical Congress will convene in Havana, in January, 1892. The President will be Dr. Juan Santos Fernandez. The congress was organized two years ago under very auspicious circumstances.

